# Repetitive transcranial magnetic stimulation promotes motor function recovery in mice after spinal cord injury via regulation of the Cx43-autophagy loop

**DOI:** 10.1186/s13018-024-04879-6

**Published:** 2024-07-02

**Authors:** Lechi Zhang, Zhihang Xiao, Zelin Su, Xinlong Wang, Huifang Tian, Min Su

**Affiliations:** 1 Department of Rehabilitation Medicine, The Fourth Affiliated Hospital of Soochow University, Suzhou, China; 2 Institute of Rehabilitation, Soochow University, Suzhou, China; 3 Rehabilitation Medicine Center of Suzhou Hospital Affiliated to Nanjing Medical University, Suzhou, China

**Keywords:** Spinal cord injury, rTMS, Neuromodulation, Motor function, Astrocyte, Connexin43, Autophagy

## Abstract

Spinal cord injury (SCI) is a severe condition with an extremely high disability rate. It is mainly manifested as the loss of motor, sensory and autonomic nerve functions below the injury site. High-frequency transcranial magnetic stimulation, a recently developed neuromodulation method, can increase motor function in mice with spinal cord injury. This study aimed to explore the possible mechanism by which transcranial magnetic stimulation (TMS) restores motor function after SCI. A complete T8 transection model of the spinal cord was established in mice, and the mice were treated daily with 15 Hz high-frequency transcranial magnetic stimulation. The BMS was used to evaluate the motor function of the mice after SCI. Western blotting and immunofluorescence were used to detect the expression of Connexin43 (CX43) and autophagy-related proteins in vivo and in vitro, and correlation analysis was performed to study the relationships among autophagy, CX43 and motor function recovery after SCI in mice. Western blotting was used to observe the effect of magnetic stimulation on the expression of mTOR pathway members. In the control group, the expression of CX43 was significantly decreased, and the expression of microtubule-associated protein 1 A/1b light chain 3 (LC3II) and P62 was significantly increased after 4 weeks of spinal cord transection. After high-frequency magnetic stimulation, the level of CX43 decreased, and the levels of LC3II and P62 increased in primary astrocytes. The BMS of the magnetic stimulation group was greater than that of the control group. High-frequency magnetic stimulation can inhibit the expression of CX43, which negatively regulates autophagic flux. HF-rTMS increased the expression levels of mTOR, p-mTOR and p-S6. Our experiments showed that rTMS can restore hindlimb motor function in mice after spinal cord injury via regulation of the Cx43-autophagy loop and activation of the mTOR signalling pathway.

## Introduction

Spinal cord injury (SCI) refers to temporary or permanent changes in the spinal cord. It has the characteristics of high morbidity, high cost and high disability [1]. SCI can be caused by high-intensity trauma, such as traffic accidents, falls, or violent injuries, or by infection, tumours, degenerative spinal diseases or vascular causes. Severe spinal cord injury places great physical, psychological and economic burdens on patients and their families [2]. However, there are currently no effective therapeutic options for SCI in the clinical setting. Indeed, the precise mechanism through which astrocytes are injured in SCI is largely unclear.

Repetitive transcranial magnetic stimulation (rTMS), a noninvasive technology, has obvious therapeutic potential for treating SCI [3]. The principle of rTMS for the treatment of SCI is rapid electromagnetic conversion. During rTMS treatment, nerve cells cause a series of physiological and biochemical reactions. The reactions are influenced by various stimulatory parameters, such as intensity, frequency and site, which could also guide therapeutic strategies [4]. In Parkinson’s disease, motor function can be improved by rTMS [5]. In the treatment of depression with rTMS, the p11/BDNF/Homer1a signalling pathway is involved [6]; and in vascular dementia, autophagic flux is activated by rTMS [7]. However, the molecular mechanisms underlying the effects of rTMS-based therapy on SCI are not well understood. Therefore, this study aimed to observe the therapeutic effects of rTMS on SCI and the related mechanisms.

Autophagy is a major catabolic pathway for the cellular degradation and recycling of macromolecules and organelles in mammals. Its main role is to remove damaged organelles, degrade proteins, and reestablish intracellular metabolism to maintain intracellular homeostasis and health [8]. Autophagic flux is impaired after SCI, and the dynamic balance of autophagic flow is disrupted, leading to autophagy stress and the accumulation of numerous oxidative stress products to accelerate cell apoptosis [9]. Recent studies have demonstrated that gap junction protein 43 (connexin43, Cx43) protects embryos from reactive oxygen species (ROS)-induced autophagic stress and apoptosis, maintaining the mitochondrial membrane potential and production of ATP [10], while baclofen can enhance protective autophagy [11] by downregulating the expression of Cx43 on cells and mitochondrial surfaces. These findings indicate that Cx43 regulates autophagy during oxidative stress.

After complete transection of the adult mouse spinal cord, the expression levels of Cx43 mRNA and protein on astrocytes are increased within a few hours, especially in the grey matter rostra at 4 weeks after injury, reaching more than three times the normal levels [12]. Cx43 is the basic functional protein of gap junctions (GJs)/hemichannels (HDs) and plays an important role in information transfer and material transport between astrocytes and neurons. Several studies have shown that the downregulation of Cx43 expression by a Cx43 inhibitor or other drugs can reduce cerebral infarction volume, protect neurons, and promote neurological function recovery [13]. Studies have shown that magnetic stimulation can affect a variety of proteins on and outside the membrane, which can affect the normal physiological functions of cell membrane receptors, enzymes and ion channels, as well as DNA synthesis, RNA transcription and cell proliferation [14]. However, how does rTMS affect Cx43 levels after SCI? How will it affect astrocytes after SCI? To date, no reports on this topic exist. This paper intends to conduct further in-depth research on this topic.

## Materials and methods

### Animals and surgeries

All animal procedures were approved by the Suzhou Institute of Biomedical Engineering and Technology Guidelines for the Care and Use of Experimental Animals. All male C57BL/6J mice (6–8 weeks, 22–26 g) were housed in a specific pathogen–free animal facility with free access to food and water. The mice were divided into four groups: (1) the normal group, (2) the SCI group, (3) the sham-rTMS group, and (4) the rTMS group. The latter three groups of mice were subjected to surgery at the age of 8–10 weeks and anaesthetized with pentobarbital (40 mg/kg, i.p.). Afterwards, complete spinal T10 transection was performed. During this operation, a needle was pressed against the lateral and ventral sides of the vertebral cavity for completeness of the lesion. Finally, urine was collected twice daily until the mice were sacrificed. The operator was blinded to the group allocation.

### Repetitive transcranial magnetic stimulation

The rTMS equipment (MagNeuro, VISHEE, China) was connected to a 100-mm outer wing diameter that was a figure-of-eight coil. The rTMS intervention began 48 h after modelling. To ensure effectiveness, the mice were placed in plastic containers after waking up from surgery, and the electrical equipment in the room was minimized. Then, the heads of the mice were placed under the centre of the coil. The resting motor threshold (RMT), the minimum stimulus intensity that elicits a hind limb twitch, was determined by monitoring the muscle contraction elicited by a single TMS pulse. The minimum stimulation intensity output (100% RMT) was used as the reference during the treatment after surgery [15]. This was typically approximately 40% of the maximum stimulus output. Each experimental animal received a daily HF-rTMS session consisting of 2400 pulses. The parameters were as follows: 15 Hz, 80% RMT, 10 pulses per string, 20 s intervals, 20 strings each time, once a day and six courses a week for 6 weeks. The mice in the sham-rTMS group were exposed to the device, but the head was perpendicular to the coil (Fig. 3A).

### Motor evoked potential (MEP) test

The MEP was measured in each mouse 6 weeks after SCI. Briefly, the mice were anaesthetized. Needle electrodes were used for both stimulating and receiving electrodes. The stimulating electrode was inserted under the skull into the motor cortex, and the recording electrode was placed on the tibialis anterior muscle. The motor cortex was stimulated with the BIOPAC MP150 system (Biopac Systems Inc., USA), the stimulation intensity was 9 V, the stimulation frequency was 1 Hz, the stimulation pulse width was 0.2 ms, and the MEP signals of the right hind limbs were recorded using Biopac Acknowledge 4.3 software. We continuously recorded the amplitude of the MEP 20 times.

### Cell culture

#### Isolation and culture of primary astrocytes

Foetal mice (gestational age 14–16 days) were obtained, and the spinal cord was dissected under a microscope and placed in a small glass Petri dish (with PBS). After the surface capsule of the spinal cord was stripped under a microscope, the spinal cord tissue was cut into pieces, 0.25% trypsin was added, and the tissue was digested for 15–20 min. The digested tissue was mixed with a pipette, filtered and centrifuged for 5 min, after which the supernatant was discarded. The cells were then resuspended in PBS and centrifuged for 5 min, after which the supernatant was discarded. The tissues were added to DMEM containing FBS, which stopped the digestion process. The solution was allowed to rest for 10 min (the termination time could be extended) until the small granular tissue on the tube wall had completely settled before mixing. After mixing the tissue solution with a pipette, filtration and centrifugation were performed, and the solution was added to cell culture plates coated with poly-L-lysine in culture flasks or Petri dishes one day ahead of time. The plates were placed in an incubator, and the solution was changed every three days. The plates were removed to determine the cell density under a microscope.

### Protocol for magnetic stimulation of astrocytes

The rTMS device was connected to a standard 70 mm outer wing diameter double, which was a circular coil with a frequency of 15 Hz [16]. Approximately 2 × 10^5^ cells were included in each dish. Astrocytes were randomly divided into two groups: (1) the sham stimulation group and (2) the magnetic stimulation group. The Petri dish was placed below the coil at a distance of 1.0 cm from the dish. The stimuli consisted of 20 strings of 100 pulses at 15 Hz (2 s per string) with a stimulus intensity of 0.5 T and a 58 s interval between each string to allow effective cooling of the coil. rTMS was applied for 20 min once a day for 7 consecutive days. The normal group did not receive magnetic stimulation, and the sham group cells were exposed to the device without active stimulation.

### Immunohistochemistry

The cells were fixed with absolute methanol for 20 min, permeabilized with 0.1% Triton X-100 for 20 min, and blocked for 1 h in 0.1% phosphate buffer (PBS)-Tween containing 1% bovine serum albumin (BSA), 10% FBS, and 0.3 M glycine. Primary antibodies against Cx43, 1:400, CST 104,224; LC3II, 1:300, CST, 43,566; P62, CST, 23,214; 1:300) were added and incubated overnight, and secondary antibodies (Alexa Cy3-conjugated anti-mouse IgG, 1:500) were subsequently added. The cells were visualized under a Zeiss LSM900 confocal microscope and quantified by ImageJ (NIH). At least 60 cells per group were analysed.

Mice were sacrificed in the sixth week after SCI and then perfused with 4% paraformaldehyde overnight at 4 °C. The spinal cord segments were transferred to 20% sucrose for 8 h and 30% sucrose for another 18 h, after which the tissues around the lesion were embedded in OCT. Then, the tissues were sectioned (30 μm) and incubated overnight at 4 °C with primary antibodies (GFAP, Abcam, 7260; Cx43, CST, 104,224; LC3II, CST, 43,566; P62, CST, 23,214; p-S6, Cell Signaling, 2215; p-mTOR, 5536; mTOR, 2983, Cell Signaling; Dapi, Sigma, D9542) overnight at 4 °C and then with Alexa 488- or 555-conjugated secondary antibodies (Abcam, ab150081; ab150110) overnight at 4 °C.

### Western blotting

The length of the spinal cord was approximately 1 mm, and the lesion scar was located. Spinal cord and cell samples were subjected to sodium dodecyl sulfate–polyacrylamide gel electrophoresis (SDS‒PAGE) and transferred to polyvinylidene difluoride (PVDF) membranes. The blots were incubated with primary antibodies and secondary antibodies overnight at 4 °C. After that, the blots were developed with enhanced chemiluminescence (ECL) and visualized using ImageLab™ software.

### Behavioural analysis

The Basso Mouse Scale (BMS) open field locomotion rating scale was used to assess locomotion from pre-operation to 4 weeks postinjury. This process was evaluated by two researchers who were blinded to the group allocation.

### Statistical analysis

The data are shown as the means ± SEMs and were analysed using GraphPad Prism 8.01. Student’s t test was used for single comparisons between two groups. The BMSs were analysed using two-way ANOVA followed by Dunnett’s multiple comparison test. Other data were analysed using one-way ANOVA followed by Dunnett’s multiple comparison test. ^*^P < 0.05, ^**^P < 0.01, ^***^P < 0.001, or no significant difference (NS) denote the significance thresholds.

## Results

### HF-rTMS inhibited Cx43 expression and negatively regulated autophagic flux in astrocytes

**Fig. 1:**
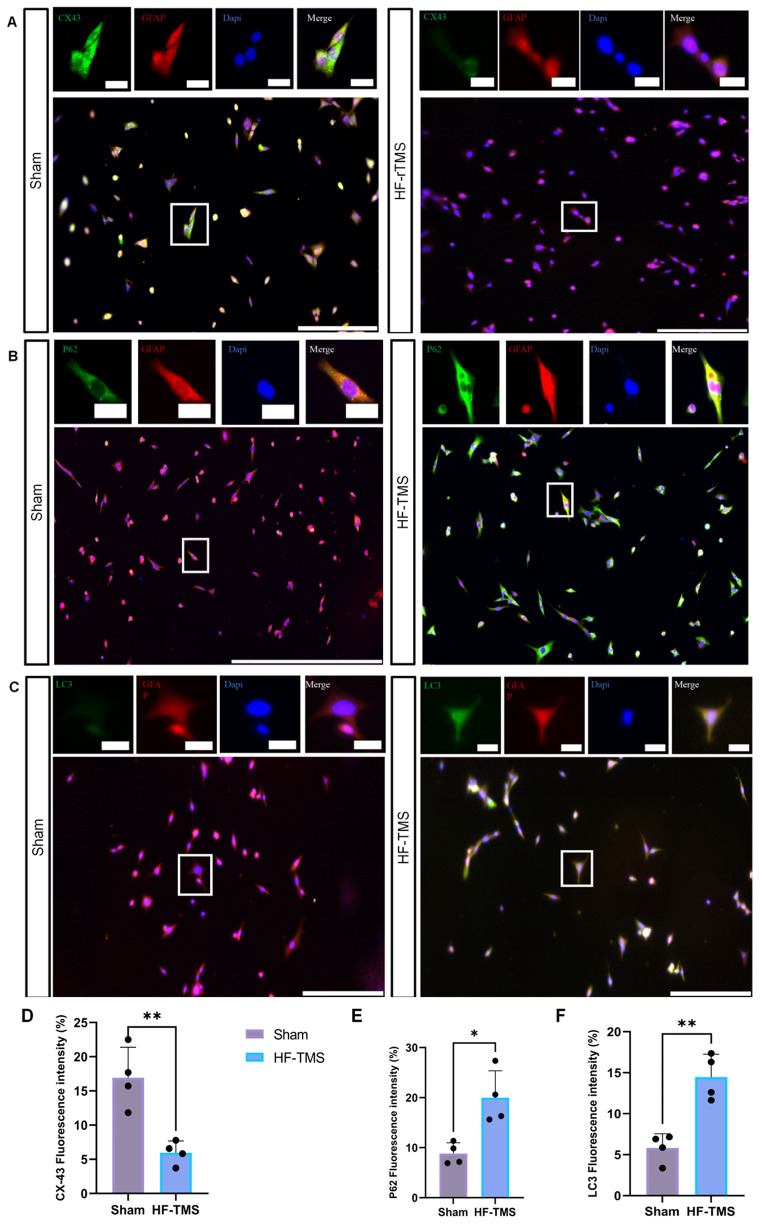
**A**. Changes in Cx43 immunofluorescence in astrocytes before and after magnetic stimulation. Its expression was downregulated in the HF-TMS group. **B**, **C**. Changes in the expression of autophagy markers (LC3 and P62) in astrocytes before and after magnetic stimulation. Scale bars, 200 μm. **D**, **E**, and **F**. Analysis of the mean fluorescence intensity of CX43, LC3, and P62. **P < 0.01, ***P < 0.005; one-way ANOVA followed by Dunnett’s multiple test. *n* = 6 mice per group

**Fig. 2:**
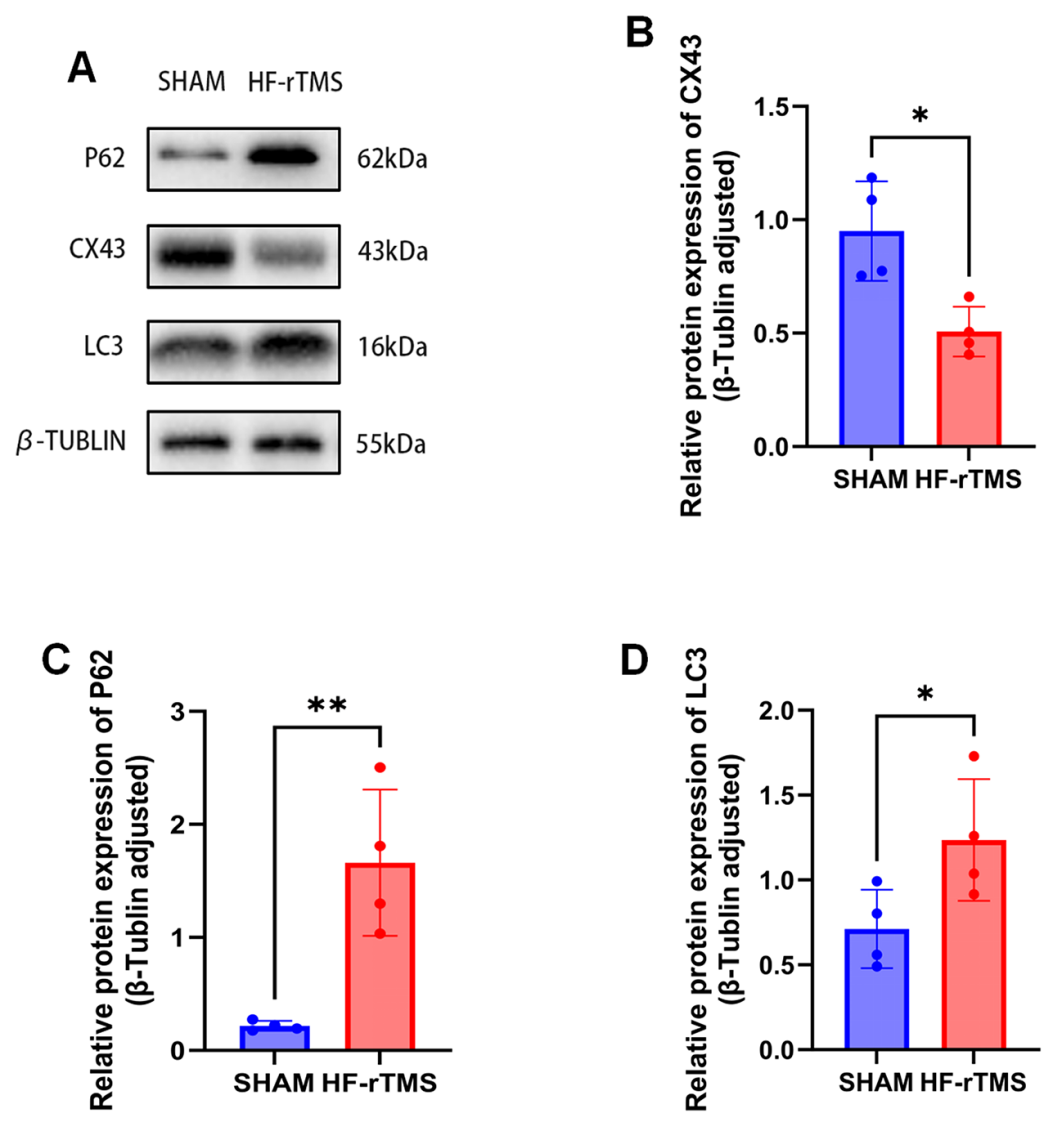
**A. B. C. D.** Western blot analysis and quantification of the levels of Cx43, P62 and LC3 in astrocytes before and after magnetic stimulation. One-way ANOVA followed by Dunnett’s multiple range test was used. *P < 0.05, **P < 0.01. *n* = 4 mice per group

CX43 is expressed mainly on the membrane of astrocytes in the nervous system, so we investigated the effect of high-frequency repetitive transcranial magnetic stimulation on the expression of CX43 on primary astrocytes. Studies have shown that HF-rTMS significantly reduces Cx43 expression on astrocytes. To verify the relationship between autophagy flux and autophagy, immunofluorescence and western blotting were used to determine the levels of autophagy markers (LC3 and P62). (Figures 1B and C and 2A) The results showed that the LC3II and p62 expression levels were significantly increased in astrocytes after HF-rTMS treatment, indicating that Cx43 may play a role in regulating autophagic flux.

### HF-rTMS increased the BMS and improved electrophysiological parameters after SCI

**Fig. 3:**
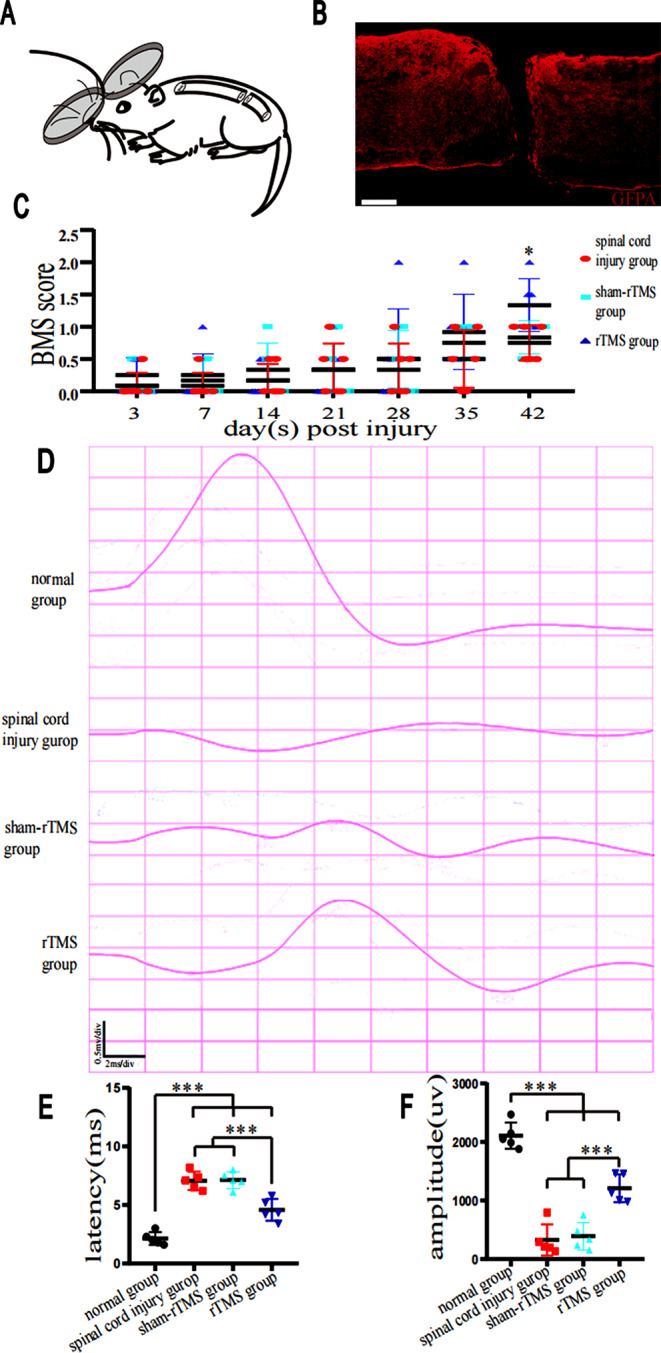
Changes in the BMSs and electrophysiological indicators of SCI model mice after rTMS. **A**, Diagram showing rTMS-treated mice after SCI. **B**, GFAP-positive astrocytes at lesion borders in mice after SCI. Scale bar = 400 μm. **C**, Time course of the change in the BMS after complete spinal cord transection. *P < 0.05; two-way ANOVA followed by Dunnett’s multiple test. *n* = 9 mice per group. **D**, MEPs in the four groups were recorded, and electrophysiological responses were recorded at 28 days postinjury. **E**-**F**, The latencies and amplitudes were analysed and quantified in each group. *P < 0.05, **P < 0.01 and ***P < 0.001; one-way ANOVA followed by Dunnett’s multiple test. *n* = 5 mice per group

We used glial fibrillary acidic protein (GFAP), a marker of astrocytes, to outline the borders of the lesion (Fig. 3B). GFAP staining showed that our model was successfully constructed. In this study, locomotor function in the open field test was measured by the BMS. Among the SCI group mice, sham group mice and rTMS group mice, hindlimb stretching function was severely impaired for the first week after injury and slightly improved over time. Notably, the mice in the rTMS group had greater BMSs than those in the sham-SCI group at 28 days postinjury (*P* < 0.05) (Fig. 3C). To further evaluate the treatment effect, electrophysiological transmission was measured at 28 days after injury. Compared with those of the normal group mice, the latencies of the SCI group mice were prolonged, and the amplitudes of the latencies were significantly reduced (*P* < 0.001) (Fig. 3D-F). In contrast, evoked responses were restored in the rTMS group. The latencies were decreased, and the amplitudes were significantly increased (*P* < 0.05) (Fig. 3D-F).

### HF-rTMS inhibited the expression of CX43 in the scar after spinal cord injury and negatively regulated autophagic flux in mice

**Fig. 4:**
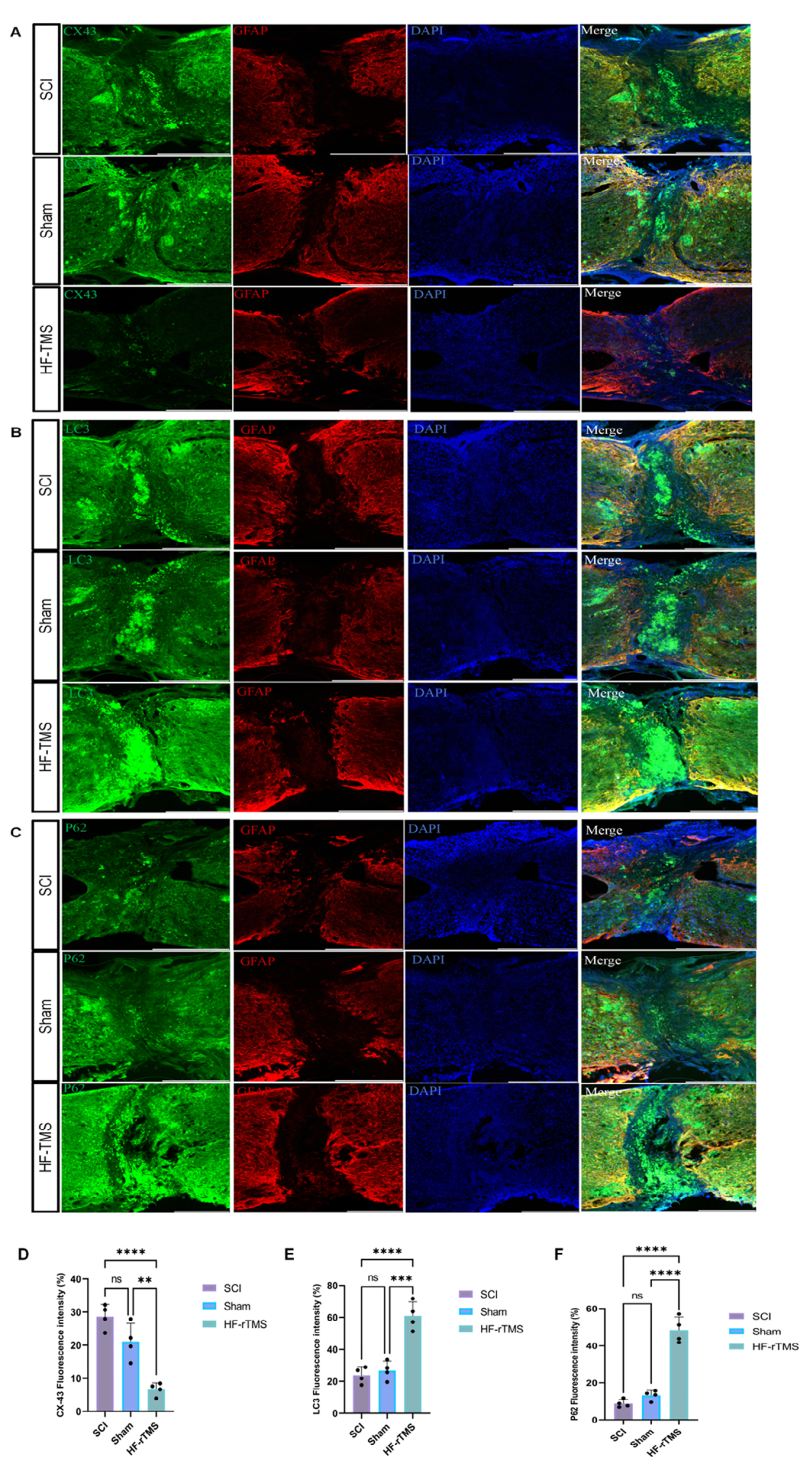
**A**. Changes in the expression of Cx43 by immunofluorescence in the lesion area of the spinal cord before and after magnetic stimulation. Its expression was downregulated in the HF-TMS group. **B**, **C**. Changes in the expression of autophagy markers (LC3 and P62) in the lesion area before and after magnetic stimulation. Scale bars, 500 μm. **D**, **E**, and **F**. Analysis of the mean fluorescence intensity of CX43, LC3, and P62 (*n* = 6). **P < 0.01, ***P < 0.005; one-way ANOVA followed by Dunnett’s multiple range test

**Fig. 5:**
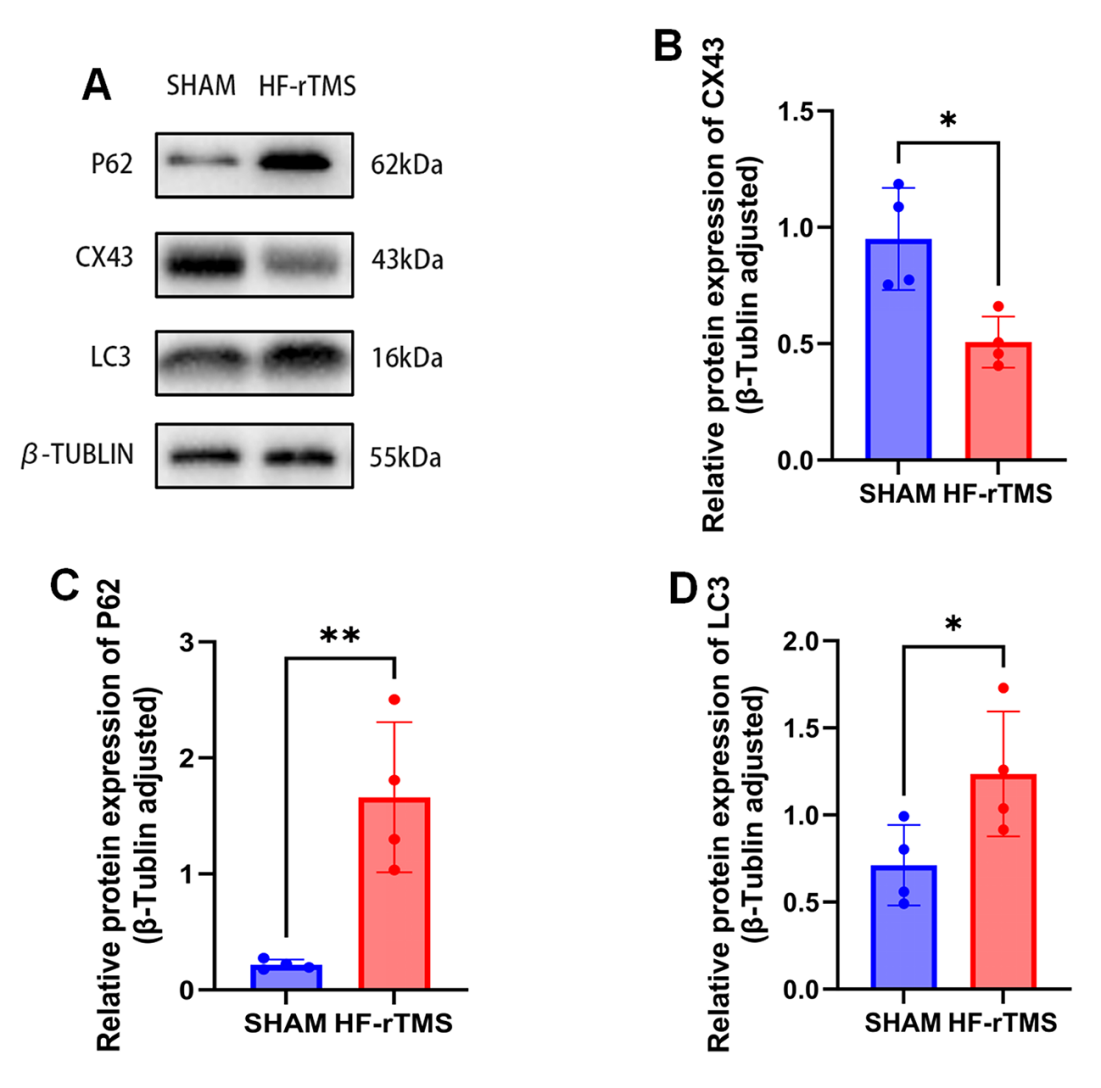
**A. B. C. D.** Western blot analysis and quantification of the levels of Cx43, P62 and LC3 in the lesion area before and after magnetic stimulation. One-way ANOVA followed by Dunnett’s multiple range test was used. *P < 0.05, **P < 0.01. *n* = 4 mice per group

To investigate the relationship between HF-rTMS and Cx43 and autophagy in mice with spinal cord injury (SCI). Immunofluorescence and Western blot analysis of Cx43, LC3, and P62 were performed on the scar tissue from the injured spinal cord. Notably, the expression of Cx43 in the spinal cords of SCI model mice treated with HF-rTMS was significantly lower than that in the spinal cords of sham-treated mice (Figs. 3A and 4A). However, the levels of LC3 and P62 were significantly increased (Figs. 3B and C and 4A). Thus, Cx43 inhibition increases autophagic flux in the spinal cords of SCI model mice (Fig. 5).

### HF-rTMS can regulate autophagic flux in the spinal cord of SCI mice by enhancing the mTOR signalling pathway

**Fig. 6:**
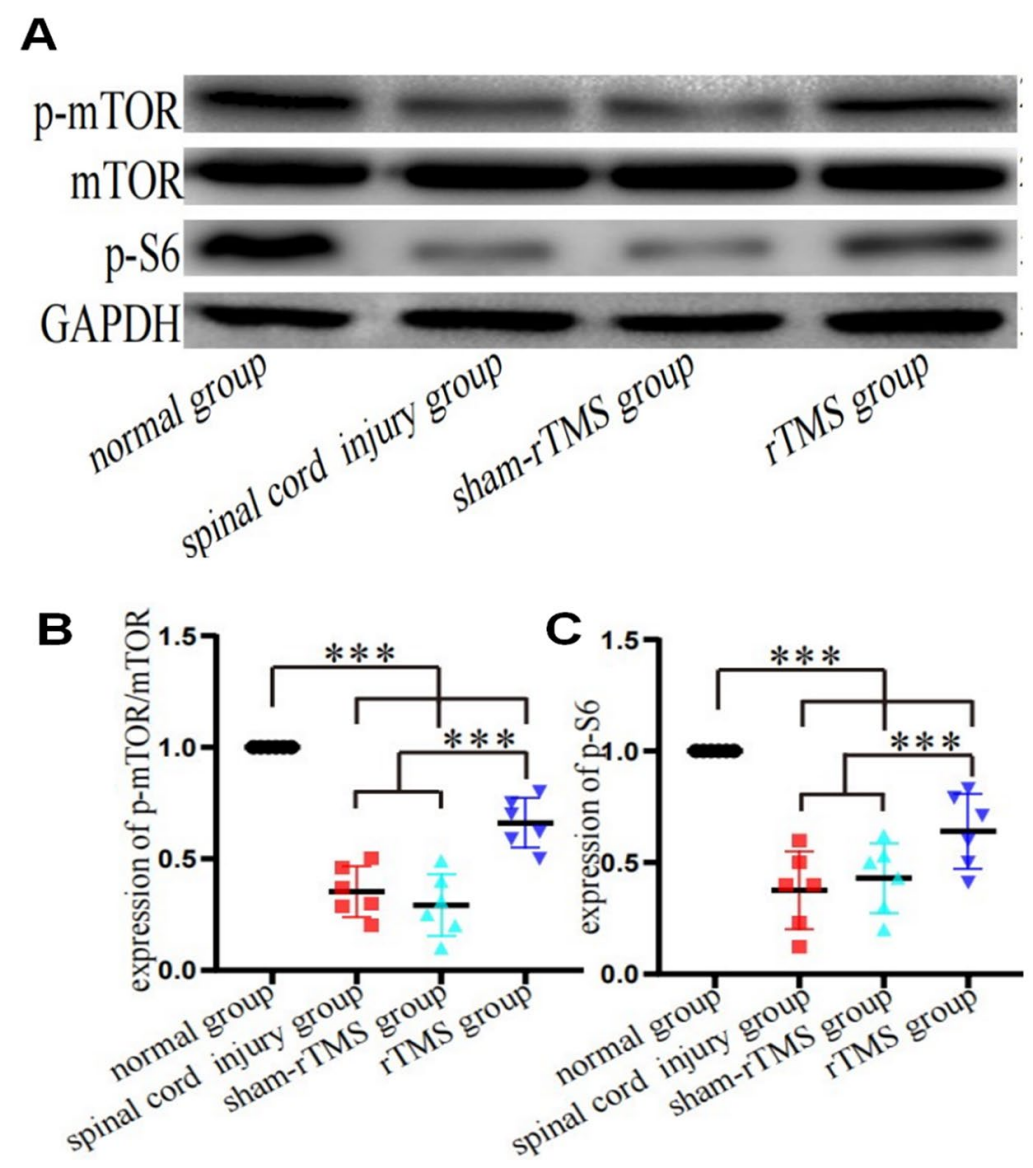
rTMS increased the level of mTOR after SCI. **A. B. C**. Western blot analysis and quantification of the levels of p-mTOR/mTOR and p-S6 in the lesion area at 28 days postinjury. *P < 0.05 and ***P < 0.001; one-way ANOVA followed by Dunnett’s multiple range test. *n* = 6 mice per group

To investigate whether mTOR signalling is involved in the regulation of autophagic flux by HF-rTMS in vivo, we measured the level of mTOR. Western blotting revealed that the p-mTOR/mTOR protein ratio was significantly increased in the spinal cords of mice with SCI after HF-rTMS treatment (Fig. 6A B and C). Therefore, activation of the mTOR pathway may negatively promote the autophagic flow regulated by Cx43.

## Discussion

Little is known about the molecular signals involved in functional recovery after SCI. In this study, we observed that motor function increased and electrophysiological indicators improved after HF-rTMS. However, the mechanism by which HF-rTMS ameliorates spinal cord injury is unclear. Impaired autophagy appears to play a key role in the course of spinal cord injury [17]. Recently, Cx43 has been reported to be involved in the process of autophagy [18, 19]. However, whether Cx43 plays a role in autophagy in SCI remains largely unknown. The present study suggested that HF-rTMS can decrease Cx43 expression in astrocytes and may negatively regulate autophagic flux. Inhibition of Cx43 expression can improve impaired autophagy flux and promote motor function and electrical conduction in the corticospinal tracts of SCI model mice by activating the mTOR pathway. These findings may help to further the understanding of the mechanism of magnetic stimulation therapy for spinal cord injury and provide new therapeutic targets for treating spinal cord injury.

Autophagy (“self-feeding”) is a tightly regulated process that delivers senescent intracellular components to lysosomes for degradation to maintain intracellular homeostasis [20, 21]. Recent studies have shown that autophagy regulation is closely related to human health [22]. After SCI, an inhibitory microenvironment is formed in the lesion area, and adult neurons exhibit poor intrinsic growth potential [23, 24]. After SCI, autophagic flux is impaired, and the accumulation of a large number of oxidative stress products accelerates apoptosis [10, 11]. Furthermore, Cx43 has been shown to protect embryos from reactive oxygen species (ROS)-induced autophagic stress and apoptosis to ensure the maintenance of mitochondrial membrane potential and ATP production, while baclofen can promote protective autophagy by downregulating Cx43 expression on cell and mitochondrial membranes [25]. These results suggest that the regulation of Cx43 and autophagy is closely related to the process of oxidative stress and that the relationship between Cx43 and autophagy flux after SCI may strongly affect recovery after SCI. The in vitro and in vivo data of the present study suggest that Cx43 expression is significantly elevated after SCI, which may contribute to further development of SCI. Interestingly, after HF-rTMS intervention, the expression of Cx43 in astrocytes and spinal cord injury sites was significantly inhibited, while the impaired autophagy flux was significantly abrogated, indicating that Cx43 is partially involved in the pathogenesis and progression of SCI. Targeting impaired autophagy flux represents a new mechanism of rTMS that mediates the effect of Cx43 on SCI. However, the exact regulatory mechanism of rTMS on the Cx43-autophagy loop has not been fully elucidated, and more studies are still needed to clarify this mechanism.

The main principle of rTMS technology is to use a time-varying current to produce a time-varying intensity pulse when it passes through a coil. Magnetic pulses can produce spatially distributed voltage differences and stimulate central nervous system (CNS) tissue in the body [26]. In animal models of stroke, rTMS technology can promote nerve sprouting and synaptic remodelling, extend the information processing of the cerebral cortex and related distant areas, and adjust the time course of neural reflexes, thus enhancing the function of the neural network and ultimately improving the clinical symptoms of the model animals [27]. SCI can trigger the activation and proliferation of astrocytes, resulting in changes in their phenotype and morphology. Previous studies [28] suggested that the activation and proliferation of astrocytes could inhibit axon regeneration and hinder the recovery of nerve function during injured spinal cord repair. Recently, it has been reported that the early application of magnetic stimulation can inhibit the activation of astrocytes, reduce the formation of glial scars in the spinal cord injury area, and promote the recovery of motor function [29]. More importantly, TMS can inhibit the autophagic activity of neurons in the penumbra region of infarcted rats and promote the recovery of neurological function after cerebral infarction [30]. This finding is similar to the results of our study.

mTOR is a well-known biological factor that regulates cell metabolism, growth, and proliferation in response to multiple triggers. Deregulation of mTOR signalling, which plays a key role in regulating autophagy, has been reported in many human diseases, including diabetes, neurodegenerative diseases, and cancer [31]. A study revealed that the mTOR signalling pathway could promote axon regeneration [32]. Electrophysiological techniques further demonstrated that the activation of mTOR facilitates the transmission of information. These results proved that rTMS increased the expression of mTOR and promoted functional recovery.

Molecular biology experiments have proven that rTMS can regulate synaptic plasticity. In the external environment, synaptic plasticity occurs, and neurons may undergo adaptive changes to maintain their relative stability and synaptic transmission efficiency [33, 34]. After SCI, synapses are damaged due to direct injury, the release of inflammatory factors, and the induction of oxidative stress [35, 36]. Studies have shown that regulating synaptic plasticity can significantly alleviate dysfunction after SCI [37]. In the future, we will explore the mechanism by which rTMS regulates synaptic plasticity after SCI.

In conclusion, this study identified and revealed a previously unknown underlying mechanism by which TMS improves SCI. Studies have confirmed that HF-rTMS can inhibit the expression of Cx43 on the membrane of astrocytes and negatively regulate autophagy by activating the mTOR signalling pathway in spinal cord tissue to promote recovery after SCI. Therefore, the Cx43-autophagy loop may be a mechanism by which rTMS improves neurological function.

## Data Availability

The data presented in this article can be obtained from the corresponding author upon reasonable request.
